# Endogenous Endophthalmitis: A Five-year Review of Cases at the Raja Perempuan Zainab II Hospital, Kelantan, Malaysia

**DOI:** 10.7759/cureus.3066

**Published:** 2018-07-30

**Authors:** Ngu Dau Bing Michael, Shakiran Gunaseelan, Tengku Norina Tuan Jaffar, Zamri Noordin, Adil Hussein

**Affiliations:** 1 Ophthalmology Department, School of Medical Sciences/Universiti Sains Malaysia, Kelantan, MYS; 2 Ophthalmology Department, Raja Perempuan Zainab Il Hospital, Kelantan, MYS

**Keywords:** endogenous endophthalmitis, vitrectomy, intravitreal injection, bacteria

## Abstract

Background

Endogenous endophthalmitis (EE) is a type of intraocular inflammation secondary to hematogenous spread from a distant infective source within the body and usually occurs in immunocompromised patients.

Objectives

The aim of this study was to profile the patient characteristics, sources of infection, microbial profiles, and visual outcomes of patients with EE in Raja Perempuan Zainab II Hospital in Kelantan, Malaysia.

Materials and methods

Data from 18 eyes of 17 patients diagnosed with EE and admitted to the eye ward of Raja Perempuan Zainab II Hospital from January 2012 to December 2016 were retrospectively reviewed. Factors analyzed included patient age, sources of infection, visual acuity, microbial profiles, and treatment outcomes.

Results

The mean age of the 17 patients was 53.2 years. Twelve patients (70.6%) had EE of left eye, four (23.5%) had EE of right eye, and one (5.9%) had EE involving both the eyes. Sixteen patients (91.1%) had at least one predisposing condition, the most common of which was diabetes mellitus in 15 patients (88.2%). A source of infection was identified in 12 of the 17 patients, with urinary tract infection being the most common (five patients, 29.4%). Organisms were successfully isolated from 10 (58.8%) patients, including seven (41.2%) with Gram-negative and three (17.6%) with Gram-positive organisms. All patients presented with a visual acuity worse than 6/60. Nine (52.9%) patients underwent vitrectomy, with only two of these patients achieving a final visual acuity better than 6/60. Eleven patients became nonperceptive to light, with four of them undergoing evisceration.

Conclusions

EE is a rare but often devastating ocular condition. Visual outcomes are often poor especially in patients infected with Gram-negative bacteria.

## Introduction

Endogenous endophthalmitis (EE) is a fulminant ocular infection resulting in blindness. It is caused by hematogenous spread from a distant infective source within the body and usually occurs in immunocompromised patients or those with prolonged indwelling medical devices. EE is relatively rare, accounting for only two percent to eight percent of patients with endophthalmitis [1].

Under normal circumstances, the blood ocular barrier (BOB) provides a natural resistance against invading organisms. In EE, the BOB is breached by inflammation, and the microorganisms can penetrate through this barrier into the uveal tract or retinal circulation. This inflammatory process results in the destruction of intraocular tissues [2]. EE is associated with an extremely poor prognosis in most patients. This study aimed to describe the demographic characteristics, microbial profiles, management, and clinical outcomes of patients with EE admitted to Raja Perempuan Zainab II Hospital in Kelantan, Malaysia.

## Materials and methods

The records of patients diagnosed with EE and managed at Raja Perempuan Zainab II Hospital from January 2012 to December 2016 were retrospectively reviewed. EE was diagnosed based on the presence of lid edema, conjunctival chemosis, corneal haziness, hypopyon, vitritis, and culture sensitivity without an external cause.

Patients with a history of ocular trauma, any ocular surgery within one year of onset, or any evidence of primary external ocular infection were excluded. Demographic characteristics, clinical presentations, microbial profiles, management, and outcomes were obtained from patients’ medical records, and data were analyzed using the IBM SPSS Statistics for Windows, Version 22.0 (IBM Corp., Armonk, NY). Data with numerical variables were described as mean and standard deviation, while categorical data were expressed by frequency (*N*) and percentage. The study protocol was approved by the National Medical Research Register Malaysia and conformed to the Declaration of Helsinki.

## Results

Data from 18 eyes of 17 patients diagnosed with EE, including 16 patients with unilateral involvement and one with bilateral involvement, were retrospectively reviewed (Table 1). The 17 patients included seven males (41.2%) and 10 females (58.8%) with a mean age 53.2 ± 13.2 years at the time of diagnosis (range, 19–70 years). Unilateral involvement of the left eye (70.6%) was more common than the unilateral involvement of the right eye (23.5%).

**Table 1:** Clinical characteristics of patients (N = 17). Abbreviations: F, female; M, male; LE, left eye; RE, right eye; BE, both eyes; DM, diabetes mellitus; HPT, hypertension; IHD, ischemic heart disease; ESRF, end stage renal failure; CKD, chronic kidney disease; NPL, non-perceptive to light; PL, perceptive to light; HM, hand movement; VA, visual acuity.

Patient	Sex (F/M)	Age (year)	Eye	Medical comorbidities	Isolate	Systemic infection	Vitrectomy (Yes/No)	Initial VA	Final VA
1	F	46	LE	DM, HPT, ESRF	Bacteria	Infected catheter	No	NPL	Evisceration
2	F	53	BE	DM, IHD	Bacteria	Pneumonia	Yes	RE NPL, LE HM	RE NPL, LE 6/18
3	M	64	RE	DM, HPT	Bacteria	Pneumonia	No	NPL	Evisceration
4	F	55	LE	HPT, Asthma	No growth	Liver abscess	Yes	1/60	6/36
5	M	61	RE	DM, HPT, ESRF, IHD	Bacteria	Pyelonephritis	No	HM	Evisceration
6	F	29	LE	NIL	Bacteria	Liver abscess	Yes	PL	NPL
7	M	57	LE	DM, HPT	Bacteria	Psoas abscess	No	PL	NPL
8	F	67	LE	DM, HPT	No growth	Pyelonephritis	No	HM	6/9
9	M	47	LE	DM, HPT, CKD	Bacteria	Unknown	No	PL	Evisceration
10	F	51	LE	DM	No growth	Pleural effusion	Yes	5/60	NPL
11	F	19	LE	DM, ESRF	No growth	Unknown	Yes	PL	NPL
12	M	70	RE	DM, HPT	Bacteria	Unknown	Yes	PL	NPL
13	M	48	LE	DM, HPT, ESRF	Bacteria	Unknown	Yes	PL	PL
14	F	59	LE	DM, HPT	Bacteria	Pyelonephritis	No	HM	HM
15	F	67	LE	DM	No growth	Pyelonephritis	No	HM	HM
16	M	52	RE	DM, HPT, IHD	No growth	Unknown	Yes	1/60	6/24
17	F	59	LE	DM, HPT	No growth	Urinary tract infection	Yes	PL	NPL

Sixteen patients (91.1%) had at least one factor predisposing to infection, the most common of which was diabetes mellitus in 15 patients (88.2%). Twelve patients (70.6%) had an identifiable source of infection, with urinary tract infection (five patients, 29.4%) being the most common. Other sources of infection included liver abscesses and pneumonia in two patients each (11.8%), and intravenous catheter, psoas abscess, and pleural effusion in one patient each (5.9%). However, sources of infection could not be identified in five patients (29.4%).

Blood or vitreous samples from 10 patients (58.8%) were culture positive, with seven (41.2%) yield with Gram-negative and three (17.6%) with Gram-positive organisms (Table 2). None of these cultures were positive for fungal infection.

**Table 2:** Microbial isolates from aqueous or vitreous samples (N = 17).

Organism	*N* (%)
Culture positive	10 (58.8)
Culture negative	7 (41.2)
Gram-positive organisms	
Staphylococcus aureus	2 (11.8)
Enterococcus species	1 (5.9)
Gram-negative organisms	
Klebsiella pneumoniae	3 (17.6)
Pseudomonas aeroginosa	3(17.6)
Enterobacter species	1 (5.9)

At presentation, all 18 eyes had a visual acuity worse than 6/60. Seven eyes (38.9%) were perceptive to light, five (27.8%) could perceive hand movements, three (16.7%) were nonperceptive to light, two (11.1%) had a visual acuity of 1/60, and one (5.6%) had a visual acuity of 5/60. All patients underwent vitreous tapping with intravitreal antibiotics, as well as being treated with systemic antibiotics once diagnosed. Only the better eye of the patient with bilateral EE was treated with intravitreal antibiotics, whereas the fellow eye was treated conservatively with topical antibiotics due to poor prognosis. Nine patients (nine eyes, 50%) underwent vitrectomy. Four patients (four eyes, 22.2%) underwent evisceration due to painful blind eye despite intravitreal antibiotics treatment. Eleven eyes of 11 patients (61.1%) had a final vision of nonperceptive to light, whereas only four eyes (22.2%) achieved functional visual acuity of 6/60 or better after treatment. Eyes infected with Gram-negative organisms showed no improvement after the treatment compared to eyes infected with Gram-positive organisms or those that were culture negative (Table 3).

**Table 3:** Visual outcomes based on microbial diagnosis (N = 17). *Excluding evisceration.

Organism	*N* (%)
Gram-positive organisms	
Improvement	1 (5.9)
No change	1 (5.9)
Deterioration*	0 (0)
Evisceration	1 (5.9)
Gram-negative organisms	
Improvement	0 (0)
No change	1 (5.9)
Deterioration*	3 (17.6)
Evisceration	3 (17.6)
Culture negative	
Improvement	3 (17.6)
No change	1 (5.9)
Deterioration*	3 (17.6)
Evisceration	0 (0)

## Discussion

Endogenous endophthalmitis is a rare devastating disease, usually occurring in older patients with underlying systemic diseases. The mean age of presentation in our study was 53.2 years, similar to the results of the systemic review, which reported that peak incidence was during the fifth decade of life [1]. A previous systemic review found that the incidence of EE was higher in the right than in left eyes, due to the more proximal and direct arterial blood flow to the right carotid artery [2]. More recent reviews, however, found that the left eye was more commonly affected suggesting that the anatomy of carotid vessels likely had little effect on the site of EE [1, 3-4]. Although many studies have reported a male preponderance [1, 3-5], our study found that women were more frequently affected.

A systemic review found that 56% of patients with EE had an underlying medical illness that predisposed to infection, with diabetes mellitus being the commonest [1], and another study reported that all patients with EE had at least one chronic systemic disease [6]. Our findings were similar, in that 88.2% of our patients had diabetes mellitus. Sources of infection were identified in most of our patients, with urinary tract infection (29.4%) being the most common. Other sources of infection included liver abscess, pneumonia, and infected intravenous catheter. 

Case series from Australia and Germany found a predominance of fungal pathogens in their patients with EE [3, 7-8]. None of the patients in our series, however, were positive for fungal pathogen. Of the 10 patients positive for bacterial infection, seven were infected with Gram-negative and three with Gram-positive organisms. This finding is consistent with studies from Japan and Singapore, which reported that Gram-negative pathogens were the main causative agents of bacterial EE in East Asians [1, 4-5]. We found that three cultures were positive for Klebsiella species and three for Pseudomonas species. A study in an East Asian population found that Klebsiella species were present in almost 90% of patients with bacterial EE [5]. Moreover, among patients with systemic Klebsiella infection, 7.3% were found to have EE caused by *Klebsiella pneumoniae* [9]. Another study found that a liver abscess in the right superior segment positive for *K. pneumoniae* infection was a significant risk factor for EE [10]. Thus, patients admitted for a liver abscess or systemic *K. pneumoniae* infection should be monitored closely for endophthalmitis.

Endogenous endophthalmitis generally results in poor functional vision, with prognosis associated with visual acuity at presentation [3-5, 8]. Patients with fungal EE were found to have a better visual prognosis than patients with bacteria EE [7-8]. Among patients with fungal EE, those infected with Candida had better outcomes than those infected with Aspergillus, likely because Aspergillus infections result in extensive retinal necrosis and choroidal damage, whereas Candida infections result only in damage to retina foci [11]. Patients infected with Gram-negative organisms usually showed a poorer prognosis than patients infected with Gram-positive organisms [1, 12]. In our series, all three patients with Klebsiella-associated EE who had a final vision of nonperceptive to light despite adequate treatment, showed that Klebsiella infection is rapidly progressive and destructive. None of our patients with Gram-negative EE showed visual improvement after treatment.

Once diagnosed with EE, vitreous tapping should be performed, and intravitreal antibiotics should be administered. Most topical, subconjunctival, and systemic antibiotics do not reach therapeutic levels within the vitreous due to the BOB [13]. This explains why patients can develop EE while on systemic antibiotics for underlying infections. Vancomycin and ceftazidime should be considered first-line intraocular antibiotics for Gram-positive and Gram-negative pathogens, respectively [14]. A 15-year review of patients with culture-positive EE in Queensland, Australia, found that microbes isolated from these patients showed 100% sensitivity to vancomycin, ceftazidime, and amikacin [15]. In addition to treating these patients with intraocular antibiotics, administration of intravenous antibiotics is also important in the management of EE to treat the underlying source of infection. If the condition does not improve or if the condition deteriorates after medical treatment, surgical intervention such as vitrectomy should be considered. Immediate vitrectomy, however, is not necessary for patients with better than light perception at presentation but may benefit patients with a visual acuity of light perception or worse; that study, however, did not include patients with EE [16]. Patients with EE who underwent vitrectomy, however, were reported to be almost three times more likely to retain useful visual acuity and more than three times less likely to require evisceration or enucleation [1], given that vitrectomy removes endotoxins, exotoxins, and microorganism [14]. All four of our patients who underwent evisceration did not undergo prior vitrectomy.

## Conclusions

Endogenous endophthalmitis is a rare but often devastating ocular condition. Visual outcomes are often poor especially in patients infected with Gram-negative bacteria. A higher index of suspicion should be maintained by physician especially when treating patients with bacteremia.
